# Nickel, palladium and rhodium induced IFN-gamma and IL-10 production as assessed by *in vitro *ELISpot-analysis in contact dermatitis patients

**DOI:** 10.1186/1471-2172-9-19

**Published:** 2008-05-15

**Authors:** Valentina Bordignon, Francesca Palamara, Paola Cordiali-Fei, Antonella Vento, Arianna Aiello, Mauro Picardo, Fabrizio Ensoli, Antonio Cristaudo

**Affiliations:** 1 Laboratory of Clinical Pathology, Department of Allergology and Laboratory of Skin Physiopathology, Institute San Gallicano, IRCCS, IFO, Rome, Italy

## Abstract

**Background:**

Recent attempts to diminish nickel use in most industrial products have led to an increasing utilization of alternative metal compounds for destinations such as the alloys used in orthopaedics, jewellery and dentistry. The present study was undertaken with the aim to evaluate the potential for an allergic response to nickel, palladium and rhodium on the basis of antigen-specific induction of inflammatory/regulatory cytokines, and to characterize, according to the cytokine profiles, the nature of simultaneous positive patch tests elicited *in vivo*.

Peripheral blood mononuclear cells (PBMC) from 40 patients with different patch test results were kept in short term cultures in the presence of optimized concentrations of NiSO_4 _× 6H_2_O, PdCl_2 _and Rh(CH_3_COO)_2_. The production of IFN-γ and IL-10 elicited by metal compounds were analyzed by the ELISpot assay.

**Results:**

We found a specific IFN-γ response by PBMC upon *in vitro *stimulation with nickel or palladium in well recognized allergic individuals. All controls with a negative patch test to a metal salt showed an *in vitro *IL-10 response and not IFN-γ production when challenged with the same compound. Interestingly, all subjects with positive patch test to both nickel and palladium (group 3) showed an *in vitro *response characterized by the release of IFN-γ after nickel stimulation and production of IL-10 in response to palladium.

**Conclusion:**

These results strongly suggest that the different cytokine profiles elicited *in vitro *reflect different immune responses which may lead to the control of the allergic responses or to symptomatic allergic contact dermatitis. The development of sensitive and specific *in vitro *assays based on the determination of the cytokine profiles in response to contact allergens may have important diagnostic and prognostic implications and may prove extremely useful in complementing the diagnostic limits of traditional patch testing.

## Background

Allergic contact dermatitis (ACD) affects a large fraction of the general population being most frequently elicited by nickel (Ni), which is responsible of a delayed-type hypersensitivity clinically revealed by the demonstration of eczematous reactions upon patch testing and by laboratory testing of antigen-specific T-cell proliferation [1-3]. These alterations are the result of the interaction between the metal ions, which act as haptens, and the skin surface, thus favouring the contact and binding with cellular and extracellular matrix proteins [4-6], and the induction of a cellular immune response against the hapten-carrier protein complex [7,8]. Recent attempts to diminish Ni use in most industrial products have led to an increase in the use of different metal compounds for specific destinations such as the alloys used in orthopaedics, jewellery and dentistry [9]. For instance, the use of palladium (Pd) increased in the past 10 years [10] also due to the fact that dentistry alloys containing Pd appear more resistant to mechanical wearing effects [11,12]. Similarly, rhodium (Rh), which is primarily used as an alloying agent to harden platinum and Pd, it is now employed in electrical material because of its low electrical and contact resistance, in jewellery (platings of silver or white gold because of its silvery white colour), in optical instruments and dental prostheses.

Nevertheless, it has been recently recognized that also exposure to metal ions such as Pd, chromium, cobalt and gold, can induce a cellular immune response and clinical manifestations of ACD, even if less frequently than Ni [13-15]. In fact, positive patch testing to PdCl_2 _and clinical manifestation of ACD to Pd have been described with increasing frequency [16,17]. Despite its low sensitization potential, also Rh has been reported as sensitizer in the form of salt [18].

In Ni sensitized subjects a simultaneous patch test reactivity to Pd has been frequently reported [19,20] and a double sensitization can be hypothesised especially in individuals wearing dental prosthesis. Nevertheless, a cutaneous cross-reactivity cannot be excluded for the chemical and structural similarities of Ni and Pd [21]. Metal ions are incomplete antigens which have to bind to endogenous peptides to be "visible" to the immune system [7] and Ni and Pd encountering the same cellular environment might form *in vivo *complexes with similar proteins thus eliciting cross-recognizing immune responses [17]. Moreover, two independent *in vitro *studies demonstrated an immunologic cross reactivity between these metals at the T cell clonal level [22,23].

Identifying both a double sensitization and a lymphocyte cross-reactivity to different metal ions *in vivo *is an important issue for clinicians in directing the choice of implant materials in order to avoid incompatible reactions. Consequently, the diagnosis of ACD to both Ni and Pd needs *in vitro *assays complementary to patch testing [24,25]. Indeed, the variability of the readouts obtained with patch testing have stimulated a number of studies aimed at developing assays characterizing the immune response to metal salts. Recently, Minang *et al*. [26] analyzed the different cytokine profiles induced by nickel in allergic or tolerant subjects by enzyme-linked immunospot (ELISpot) assay. In fact, the sensitivity and specificity of the ELISpot assay allow the measurement of very low frequencies of cytokine-producing cell, therefore offering a powerful tool for the detection and characterization of Ni sensitization with important implications for diagnostic strategies [27,28].

The present study was undertaken with the aim to evaluate the potential of the ELISpot assay in determining sensitivities to Ni or Pd and to Ni and Pd on the basis of specific induction of inflammatory/regulatory cytokines. Since all subjects were skin challenged to Ni, Pd and Rh salts, and none of them had positive skin reactivity to Rh, this metal was employed to assess the cytokine profile in non allergic subjects and to explore the possibility to identify a cellular immunity against materials with traces of heavy metals.

## Results

### Patch tests

To analyse the cytokine response to metals, we considered four different groups of subjects according to their skin responses. As summarized in Table 1, group 1 included 10 patients with a strong positive reaction to Ni; group 2 included 10 patients with strong (n = 7) or moderate (n = 3) hypersensitivity to Pd; group 3 included 10 patients showing a strong reactivity to Ni and a simultaneous weak (n = 4) or moderate (n = 6) response to Pd; and, finally, group 4 included 10 patients with negative patch testing to all metals investigated. All patients enrolled in the study (n = 40) had a negative patch test response to Rh.

**Table 1:** Characteristics of patients and healthy controls.

**Group Subject n.**	**Sex**	**Age range (median)**	**Patch test to Ni**	**Patch test to Pd**	**Patch test to Rh**
**1 n = 10**	9 F	28 – 40 (30)	9 +++	9 -	9 -
	1 M	30	1 +++	1 -	1 -
**2 n = 10**	9 F	28 – 38 (33)	9 -	7+++/2 ++	9 -
	1 M	40	1 -	1 ++	1 -
**3 n = 10**	9 F	29 – 40 (32)	9 +++	6 ++/3 +	9 -
	1 M	39	1+++	1 +	1 -
**4 n = 10**	9 F	28 – 38 (33)	9 -	9 -	9 -
	1 M	36	1 -	1 -	1 -

As described in the methods, patch test reactivity to the different metal salts was defined as strong (+++: oedema, erythema, papules and vesicles), moderate (++: oedema, erythema and papules), weak (+: oedema and erythema) or no reaction (-).

### IFN-γ and IL-10 production by PBMC in response to Ni, Pd and Rh

To determine the production of IFN-γ and IL-10 upon *in vitro *stimulation, PBMC from patients with different patch test results were kept in short term cultures in the presence of NiSO_4 _× 6H_2_O, PdCl_2 _and Rh(CH_3_COO)_2 _and analyzed by the ELISpot assay.

**Figure 1:**
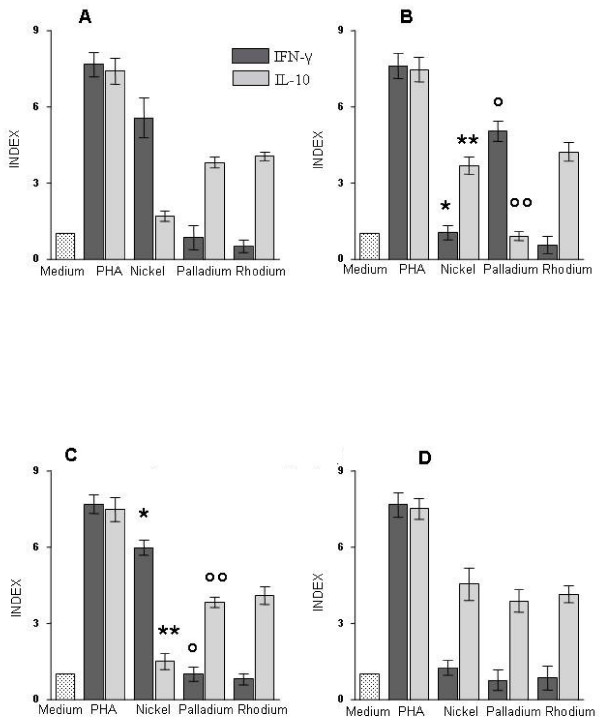
**Cytokine response elicited by metals or PHA by ELISpot analysis**. Elispot results of detection of IFN-γ or IL-10 producing PBMC upon stimulation with Ni (20 μg/ml), Pd (2.5 μg/ml), Rh (5 μg/ml) or PHA (1 μg/ml) were expressed as Index values. Index is expressed by the ratio between the number of spot forming cells (IFN-γ and IL-10 producing PBMC) upon stimulation with metal salts and those present in the absence of stimuli (spontaneous cytokine production). The mean values of Index values ± SD were obtained for the 4 different groups of 10 subjects according to their *in vivo *reactivity to metal salts. Cytokine producing cells in control triplicate wells with medium alone was the baseline value (Index = 1). Panel **A**: patch test positive to Ni (group 1); **B**: patch test positive to Pd (group 2); **C**: patch test positive to Ni and Pd (group 3); **D**: negative patch test (group 4). The statistical analysis between IFN-g and IL-10 producing cells upon stimulation with Ni or Pd between patients belonging to group C and A or B was performed using the Mann-Whitney test. The highly significant differences (P < 0.0001), found between group C and B, are indicated by symbols (*, **, °, °°).

IFN-γ and IL-10 basal response or elicited by metal salts or PHA in PBMC from different groups of patients are shown in Figure 1. The primary readouts of spot forming cells for each experimental condition in individual patients are also shown in Table 2.

**Table 2:** Individual values of spot forming cells for each experimental condition expressed as mean values and Standard Errors of triplicate wells.

			**IFN-γ**					**IL-10**				
			
	*Subject n*.	*Mean* *Std. Error*	**W/O**	**PHA**	**Ni**	**Pd**	**Rh**	**W/O**	**PHA**	**Ni**	**Pd**	**Rh**
**Patch test positive to Ni**	1	Mean	18,67	151	116	14,33	5,667	19	132	36	79,67	76
**(Group 1)**		Std. Error	2,404	4,359	3,055	1,202	0,8819	0,5774	3,606	2,082	3,93	3,786
	2	Mean	25,17	164,3	150	29,33	17,67	21	157,3	36,5	73	86,33
		Std. Error	1,74	6,766	3,215	2,333	1,453	2,082	6,36	0,2887	2,309	2,333
	3	Mean	27,67	203	136,5	19,33	16,67	11,67	92	22,5	47	52
		Std. Error	2,333	3,606	0,866	0,8819	1,453	0,8819	3,606	1,443	3,606	2,887
	4	Mean	26,67	203	168,3	18,67	5,333	33,33	270	62,67	51,33	132,7
		Std. Error	2,404	9,074	3,48	1,202	1,202	1,202	6,351	6,333	1,667	5,207
	5	Mean	30,67	246,7	168,3	27,33	27,33	38	289	65,33	143,3	147,7
		Std. Error	0,8819	1,856	2,603	0,8819	1,764	2,517	6,083	2,603	2,963	7,311
	6	Mean	13,33	100,3	53	25	6,667	22	177	32,67	79,33	93,67
		Std. Error	2,186	3,18	3,055	1,732	1,202	5,568	6,658	2,906	0,8819	4,667
	7	Mean	21,33	171,7	119,3	10,83	10,5	21,67	147,3	35,67	85,33	91
		Std. Error	0,3333	3,667	4,667	0,6009	0,2887	2,848	5,925	3,283	2,603	2,082
	8	Mean	20	158	129,3	10,33	15,5	35	265,7	46,5	136	132,7
		Std. Error	1,155	3,464	3,18	1,764	1,443	2,309	11,98	0,866	6,245	5,487
	9	Mean	20,67	160,7	118,3	4,333	8,333	33,5	233	59	129,3	130
		Std. Error	1,764	5,239	4,41	1,453	1,453	0,866	7,506	2,887	5,783	3,215
	10	Mean	13,33	105,3	65	14,67	2,667	15,5	107,7	25,33	59	62
		Std. Error	2,404	5,696	2,082	1,453	1,202	1,607	3,844	3,844	2,082	1,732

**Patch test positive to Pd**	11	Mean	10	69,33	11	45,33	2,333	27	197,3	84,33	21	129,3
**(Group 2)**		Std. Error	1,155	3,48	0,5774	2,603	1,333	2,887	7,219	2,404	3,606	3,18
	12	Mean	14,33	100,3	20,67	83,67	15,67	16,5	124,7	58,67	10,33	68,33
		Std. Error	3,844	1,453	2,333	4,631	1,202	1,443	3,283	1,202	2,603	2,906
	13	Mean	13	92	19,33	62,33	14	37,67	285	143	38	186
		Std. Error	3,215	5,686	2,028	3,844	1,528	1,453	5,508	2,309	1,732	3,464
	14	Mean	13,5	101	17	71,67	10,67	35,5	285	127,7	30	151
		Std. Error	0,866	7,937	3,215	2,333	0,8819	0,2887	5,508	4,41	1,155	1,528
	15	Mean	45	360,3	41	229,7	36,33	9,333	73,67	37,67	10,67	41,33
		Std. Error	1,732	9,351	1,732	2,906	3,48	1,764	2,728	1,202	1,764	1,856
	16	Mean	6,5	53	5,667	34	2	12	92	47,33	13,67	45,33
		Std. Error	1,443	2,082	1,202	1,155	1,528	1,528	2	1,856	1,764	1,764
	17	Mean	15,67	117,3	9,667	72,67	6,333	23,33	190	84	22	93
		Std. Error	3,48	7,688	0,6667	1,453	0,8819	2,848	2,082	2,082	2,309	1,155
	18	Mean	6,667	52	5,333	32	2	28,67	198,3	110,3	24	112
		Std. Error	1,764	3,512	0,8819	2,082	1	1,856	10,4	9,171	1,155	7,371
	19	Mean	32,67	273	36	170,3	13,33	10,33	70,33	34,33	9,667	44
		Std. Error	1,202	3,606	2,646	5,548	1,202	0,8819	2,728	3,283	1,202	2,646
	20	Mean	30,67	244	24,33	153,3	7	28,67	197,3	120,7	31,67	116
		Std. Error	1,202	4,509	2,028	2,848	1,528	2,404	2,333	8,95	1,764	4,041

**Patch test positive to Ni and Pd**	21	Mean	14	105,7	89,67	17,67	10,67	20,33	153,7	32,67	72,33	81
**(Group 3)**		Std. Error	2,646	8,293	6,692	2,404	1,764	1,453	5,783	3,528	2,333	3,786
	22	Mean	24,83	172	150	25	25	22	176	26,67	79,67	93,67
		Std. Error	2,048	3,786	3,464	2,082	2,887	5,568	6,658	3,283	2,333	2,603
	23	Mean	26,67	203,7	157	37,33	14,67	38,67	292,3	66,67	148,7	152
		Std. Error	1,453	8,686	4,619	2,333	1,764	2,186	5,783	1,856	4,055	3,606
	24	Mean	31	247	186	30,67	18,33	23,67	186,3	21,33	94,67	119,3
		Std. Error	1,155	2,309	3,464	1,453	2,028	4,702	7,623	3,528	4,41	1,856
	25	Mean	25,67	197	157,7	22,33	18	37,33	285	49	145,7	143
		Std. Error	1,764	4,041	1,856	2,028	2,309	1,453	5,508	3,215	3,383	3,215
	26	Mean	20	157,7	117	16	10	15,5	125,3	29,67	56	61,67
		Std. Error	1,155	3,18	2,082	1,155	2,517	0,2887	4,667	1,667	2,887	1,764
	27	Mean	12,33	95,67	70,33	12	9,667	30,33	210,7	39,67	114,7	118,3
		Std. Error	1,453	10,37	3,383	1,528	2,028	3,18	6,984	4,978	3,48	3,756
	28	Mean	45	361	244,3	49,67	52,67	31,67	215	50	124,7	133
		Std. Error	1,732	9,292	4,096	3,283	2,333	2,963	5,132	2,646	3,48	2,309
	29	Mean	45,5	373,3	285	60	40	38,33	264,3	72,67	161,7	153,3
		Std. Error	2,021	2,963	6,429	1,732	3,512	4,055	12,25	2,028	4,055	3,48
	30	Mean	15,67	118	96	6,333	16,33	33,67	254,3	54	132	131,3
		Std. Error	2,333	3,215	6,928	2,028	2,728	2,963	8,413	2,309	5,292	2,963

**Patch test negative**	31	Mean	3,667	24	6	4,667	4,667	32,33	243	146,7	116,7	134,3
**(Group 4)**		Std. Error	0,8819	1,528	1,155	0,8819	1,202	2,028	4,041	4,631	6,173	5,608
	32	Mean	17,33	130	27,67	21,33	33	47,33	377	237,3	181	201,3
		Std. Error	2,333	2,887	1,764	3,283	1,732	2,728	2,646	2,603	3,215	6,064
	33	Mean	6,667	53,33	10,33	4,333	5,667	18,33	140,3	69,67	66,33	90,67
		Std. Error	1,202	2,728	0,8819	1,453	0,6667	1,453	2,603	2,404	4,842	4,631
	34	Mean	11,33	82,67	15	8	8	24,33	193,3	98	99	108
		Std. Error	0,8819	2,333	2,082	1,732	1,528	1,202	3,844	1,732	4,619	5,196
	35	Mean	14,33	112,7	17,33	18,67	7,333	34,33	260,3	134	111,7	132
		Std. Error	1,202	5,044	1,453	2,028	0,8819	2,404	3,18	1,528	4,41	4,359
	36	Mean	3	23	3,333	0,6667	2,333	17	138	78,33	83	67,67
		Std. Error	0,5774	1,528	1,453	0,6667	0,3333	2,646	4,163	1,453	3,055	2,404
	37	Mean	18	141	23,33	7,333	4,333	15	109,7	57,33	57,33	58,67
		Std. Error	1,732	5,033	1,453	1,202	0,8819	1,732	2,906	2,404	3,18	4,256
	38	Mean	22,67	180,7	27,33	18,33	11,67	29	196,7	154,3	114,3	122,7
		Std. Error	1,202	1,202	2,404	1,453	1,202	1,732	1,453	2,404	6,009	4,41
	39	Mean	6,667	54	6,333	2	5,333	10	71,33	52,67	40,33	40,33
		Std. Error	1,202	2	0,8819	1,155	1,856	1,155	2,028	2,028	2,906	4,096
	40	Mean	13,33	105,3	10,67	11	15	27,67	199,3	146,3	107,7	107
		Std. Error	1,453	3,712	0,8819	1,528	1,528	1,453	3,756	11,26	4,978	7

Patients were grouped following their patch test reactions. W/O: medium alone; PHA: polyclonal stimulation; Ni 20 μg/ml, Pd 2.5 μg/ml, Rh 5 μg/ml.

PHA stimulated cultures invariably showed increased numbers of IFN-γ or IL-10 producing cells with Indexes ranging from 6.8 to 8.3, and the values did not significantly differ among the different groups, therefore excluding differences in the capability of different subjects to rise a specific cytokine response or impairment of cellular function due to cryopreservation.

Remarkably, IFN-γ releasing cells upon metal stimulation were the most represented in strict association with results of the *in vivo *responses. In fact, IFN-γ-Ni responsive cells (Index > 3) were detected in subjects with positive patch test to Ni either alone (group1) or in association with moderate or weak response to Pd (group 3) (P = 0.28). IFN-γ-Pd responsive cells were detected only in subjects with strong or moderate Pd positive patch testing (group 2), and not in subjects with moderate or weak *in vivo *response to Pd associated with a strong Ni reactivity (group 3) (P < 0.0001).

All subjects with negative patch test results showed an *in vitro *IL-10 production upon metal stimulation while any positive or ambiguous IFN-γ response to Ni, Pd or Rh was present. Interestingly a different in vitro behaviour was found between subjects showing a monosensitization to Pd and subjects with concomitant skin reactivity to Pd and Ni. In fact, these subjects (group 3) had an *in vitro *reactivity more similar to Ni monosensitized patients (IFN-γ-Ni responsive cells: P = 0.28; IL-10-Ni responsive cells: P = 0.19) rather than to Pd monosensitized subjects (IFN-γ-Ni responsive cells P < 0.0001; IL-10-Ni responsive cells: P < 0.0001).

**Figure 2:**
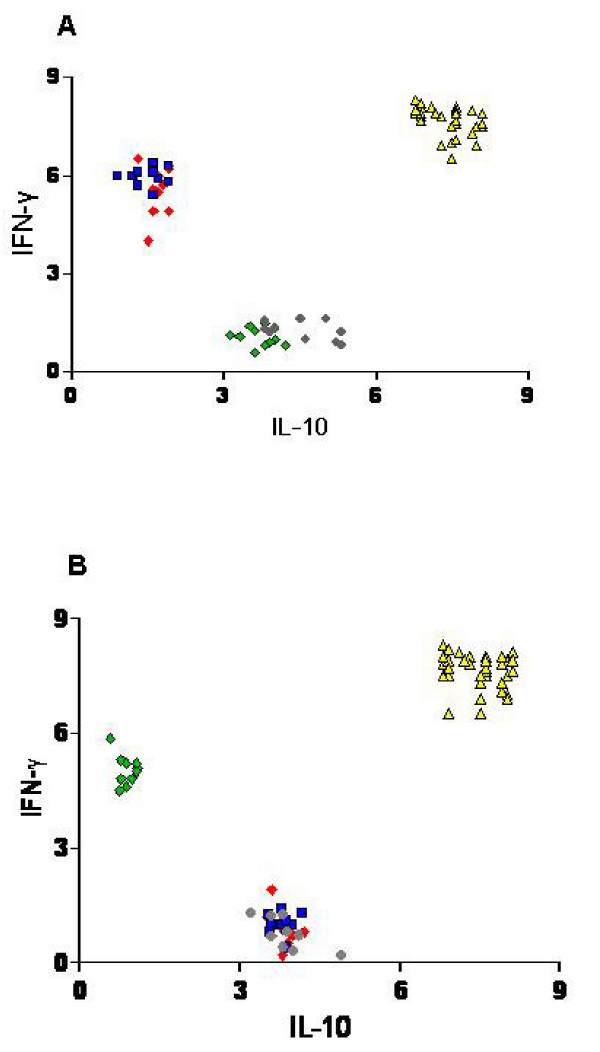
**Scatter plot analysis of individual IFN-γ and IL-10 induction by PHA, Ni or Pd in the different group of patients**. PHA induces PBMC release of both IFN-γ and IL-10 in all subjects, while metal salts induces IFN-γ or IL-10 production according to the *in vivo *positive or negative response. IFN-γ or IL-10 production was alternatively produced and consequently a highly significant negative correlation was found (Spearman analysis P < 0.0001). Panel **A**: IFN-γ and IL-10 induction (Index) by PHA or Ni salt; **B**: IFN-γ and IL-10 induction (Index) by PHA or Pd salt. *Red Diamond: Group 1 (induced by Ni in panel A or by Pd in panel B), Green diamond: Group 2 (induced by Ni in panel A or by Pd in panel B), Blue square: Group 3 (induced by Ni in panel A or by Pd in panel B), Grey circle: Group 4 (induced by Ni in panel A or by Pd in panel B), Yellow triangle: Overall subjects (induced by PHA)*.

Overall results showed that IL-10 producing cells in response to metals were invariably associated to a lack of IFN-γ production. This is well visualized by the scatter plots (Figure 2) of individual Index values obtained in response to Ni or Pd in all groups of patients, where it can be appreciated that IFN-γ or IL-10 production in response to metal salts are mutually exclusive, while PHA is capable of inducing both cytokines. Statistical analysis confirmed the significant negative correlation between IFN-γ and IL-10 production (P < 0.0001). The same diagram (Figure 2) reporting the individual responses in the different group of patients also shows that the *in vivo *moderate and weak reactivity to Pd, observed in association to strong reactivity to Ni (group 3), did not match the *in vitro *response, that was clearly sustained by IL-10 rather than IFN-γ producing cells.

## Discussion

In the present study, we have investigated the profiles of cytokine production by PBMC of ACD patients and healthy controls in response to the well known contact allergens Ni and Pd, or to Rh, considered to have a low sensitization potential, through the ELISpot assay.

The results showed that PBMC from well recognized allergic individuals affected by ACD have a higher frequency of IFN-γ releasing cells upon *in vitro *stimulation with Ni or Pd, as compared to control groups. Most interestingly, we found that PBMC from all the individuals exhibiting a negative skin response to a metal salt (group 4) revealed a clear IL-10 response when challenged with the same metal, in the absence of any detectable IFN-γ response. Accordingly, all subjects included in the study had a negative skin response to Rh, which was invariably associated to Rh-induced IL-10 production *in vitro*.

The immunologic mechanisms responsible for the clinical hypersensitivity towards metals have been recently reviewed [7]. It is known that Ni-specific, IFN-γ-producing CD4^+ ^and CD8^+ ^T cells are major effectors of sensitization and directly responsible for skin manifestations [6,29], whereas IL-10 and TGF-β-secreting regulatory T cells are thought to down-modulate the response [2,30,31]. The deleterious expansion of Ni-specific, effector CD8+ T cells might be thus prevented by regulatory mechanisms, including those induced by hapten-specific suppressor T cells [32]. In humans IL-10 has been identified as a potent anti-inflammatory cytokine produced by both Th1 and Th2 CD4+ T cells [33], which inhibits T cell proliferation of both subsets, and regulates the development of Th1 cells by blocking IL-12 driven Th1 responses [34]. IL-10 is thought to exert its effects by down-regulating the expression of co-stimulatory molecules required for appropriate antigen presentation [35]. Ferguson TA *et al *and Schwarz A *et al *[36,37] demonstrated a key regulatory role for IL-10 in both the induction and the effector phases of murine contact hypersensitivity. Our results provide evidence supporting the hypothesis that a regulatory mechanism mediated by IL-10 contributes to the control of hypersensitivity in response to Ni, Pd and Rh therefore contrasting the development of ACD. Even if the protective role of IL-10 is well established, studies addressing the cytokine profile in subjects with or without ACD have been partly contradictory [26,31,38]. It should be considered that different protocols used for the *in vitro *stimulation or the different sensitivities of the immunoassays for IL-10 measurements could be responsible for the different results obtained.

The finding that not only Ni but also other metals could elicit a response in PBMC from sensitized subjects [15], was not surprising and prompted us to explore the profile of cytokine production in patients which scored negative at patch testing toward Pd and Rh, which are present in several synthetic materials including new metallic alloys [39,40]. Remarkably, we found very clear-cut, divergent, IL-10 "protective" or IFN-γ "reactive" profiles in patients showing a negative or positive skin reactivity to metal salts (group 1, 2, 4), respectively. Moreover, all the subjects with positive patch test to both Ni and Pd (group 3) showed an *in vitro *response similar to monosensitized patients to Ni, characterized by the release of IFN-γ after Ni stimulation and production of IL-10 in response to Pd or Rh. Interestingly, comparing the cytokine response to Pd, subjects with the same patch test reactivity (n = 3: ++ in the group 2; n = 6: ++ in the group 3) displayed a reversed profile (Table 2). On the basis of these data, it is conceivable that a positive (IFN-γ) response *in vitro *may predict the development of adverse reaction to prosthesis alloys or to chemotherapeutic agents containing toxic metals. In fact, previous studies on multiple sensitivities to transition metals reported that the majority of Ni/Pd reactions occurred without clinical evidence of Pd sensitization [10,19,41].

We analyzed the IFN-γ response in patients with an unambiguous Ni patch test reaction (group 1 and 3 patients with +++ reactions) to provide positive controls for the *in vitro *assay, in order to define optimal conditions for antigen-specific cytokine production and to offer a highly sensitive and specific test suitable for clinical determinations. On the basis of this setting, stimulation values greater than 3 to 7 were commonly obtained with metal salts or with lectin (PHA) polyclonal stimulation, respectively, suggesting that cryopreservation did not affect the cytokine response of lymphocytes, as sustained by the study from Kreher *et al *[42].

The data gathered by the ELISpot analysis did not show any cross-reactivity of T-cells neither the co-existence of T cell clones with differential responsiveness. Since any Ni contamination was demonstrated in the Pd salt used for patch testing (data not shown), it is presumable that an immunologic cross-reactivity based on molecular mimicry among different hapten-carrier complexes might take place *in vivo*, at the skin level. Due to their small size, metal ions are incomplete antigens which have to bind to carrier molecules, which, in turn, are capable to capture metal ions, and shuttling this complex to the epidermal basement membrane [4,7]. On the other hand, the concomitant reactivity to Ni and Pd, observed in a number of patients by skin testing could concern also the possibility of a delayed or impaired response of skin regulatory cells. A dysfunction in T-regulatory cell can lead to an immune activation often associated with the clinical expression of skin diseases such as atopic dermatitis and psoriasis [43]. Experimental data suggested also the possibility that, in analogy to superantigens, Ni or Pd may also directly link TCR and MHC in a peptide independent manner and can effectively amplify the hapten-specific immune response [44,45]. It is also known that a strong binding of different superantigens to the TCR could render T cells insensitive to suppression by CD4^+^CD25^+ ^Treg cells [46] and IL-10 [47].

Knowledge about the immunologic mechanism responsible for ACD is still fragmentary. It is accepted that the sensitising chemical penetrates into the epidermis, and, depending on its properties, it "perturbates" the environment. However, neither the molecular nature of Ni-induced antigenic determinants nor the very early molecular events of metal transport through the human epidermis to Langerhans cells have yet been satisfactorily resolved indicating that still many aspects of metal allergy remain unclear.

## Conclusion

Taken together, these results demonstrate that different cytokine profiles elicited *in vitro *can reflect different immune responses *in vivo *in ACD. The development of sensitive and specific *in vitro *assays based on cytokine response could be extremely useful in complementing patch testing for the clinical diagnosis and characterization of metal sensitization.

## Methods

### Patients

Fourty patients (n = 36 female (f)/4 male (m); mean age 33.3 yrs, range between 28–41) attending the Department of Allergy of this Institute were enrolled in the study. Patient assessment was primarily based on a detailed medical history, including history of metal exposure. Wearing of metal dental braces was also recorded. None of the patients had recently used immunosuppressive medication, underwent UV radiation or suffered from acute inflammatory skin diseases. The study received the approval from the Internal Ethic Committee of Istituti Fisioterapici Ospitalieri (San Gallicano, IFO, Rome), and an oral informed consent was obtained from all subjects prior to the *in vitro *study.

### Patch testing

Patch testing was performed with all patients enrolled in the study. The skin patch tests were with the European standard series of contact allergens (Hermal Trolab, Reinbeck, Germany), including 5% Nickel Sulphate hexahydrate (NiSO_4 _× 6H_2_O) applied in petrolatum, and supplemented with 1% Palladium Chloride (PdCl_2_) applied in petrolatum and 1% Rhodium Acetate Rh(CH_3_COO)_2 _in aqueous solution. These salts were from Merck (AG, Darmstadt, F.R.G). The patch tests were performed by applying Finn Chambers with haptens on unaffected skin of the upper backs of the subjects for 48 h. Patch test responses were examined on day 2 and defined as strong (+++: oedema, erythema, papules and vesicles), moderate (++: oedema, erythema and papules), weak (+: oedema and erythema) or no reaction (-) according to the International Contact Dermatitis Research Group guidelines [48].

### ELISpot assay

#### Peripheral blood mononuclear cells

PBMC were isolated from 5 to 10 ml of heparinized blood, collected 48 hrs after skin testing, by standard Ficoll density-gradient centrifugation (Lympholyte-H solution Cederlane, Ontario, Canada) and washed twice with PBS. Cell aliquots were frozen in 90% heat inactivated Fetal Bovine Serum (FBS, Euroclone) and 10% DMSO (Dimethylsulphoxide, Sigma) and kept in liquid nitrogen until tested for ELISpot assay.

#### Metal salts used for in vitro cytokine assay

The metal salts used for *in vitro *assays were: nickel sulphate hexahydrate NiSO_4 _× 6H_2_O, palladium chloride (PdCl_2_) and rhodium acetate Rh(CH_3_COO)_2 _(Merck AG, Darmstadt, F.R.G). Before use, metals were resuspended in sterile saline solution at 2 mg/ml (Bioindustria, Novi Ligure, Italy). These stock solutions were found negative for LPS contamination (*Limulus *assay, BioWhittaker, Cambrex Company, USA).

#### Definition of optimal metal salt concentrations for antigen-specific cytokine production

Preliminary experiments were performed in order to determine the optimal concentration of each metal salt for IL-10 or IFN-γ producing cells induction, measured by ELISpot assay. Briefly, 3.5 × 10^5 ^PBMC/well from 5 non-allergic donors were stimulated with 10 μg/ml of phytohaemagglutinin (PHA, Sigma, Saint Louis, Missouri, USA) in the presence of different metal salts at concentrations ranging from 0.1 μg/ml to 100 μg/ml. Metal salt concentrations > 50 μg/ml, > 25 μg/ml and > 30 of Ni, Pd and Rh respectively inhibited the PHA-induced cytokine producing cells by 25%, and were considered toxic in agreement with other authors (15). Subsequent preliminary experiments showed that 20 μg/ml NiSO_4 _or 2.5 μg/ml PdCl_2 _yielded the higher frequency of IFN-γ producing cells in five subjects with clinical diagnosis of ACD caused by Ni (n = 3) or Pd (n = 2) respectively. While, PBMC from five healthy controls cultured at the same experimental conditions provided the higher frequency of IL-10 producing cells. Also the Rh(CH_3_COO)_2 _concentration of 5 μg/ml was chosen on the basis of the higher frequency of IL-10 producing cells in patients as well as controls.

#### Pre-incubation with metals

For the detection of IFN-γ, PBMC (3.5 × 10^5^/well) were pre-incubated with or without metals in round bottom polypropylene tubes (Becton Dickinson Labware) in complete medium (RPMI 1640, 10% FBS, 1% Penicillin-Streptomycin-L-Glutamine; GIBCO-BRL, UK) for 20 hrs at 37°C and 5% CO_2 _in humidified air. Then cells were transferred to the ELISpot plates and incubated for a further 48 hrs.

ELISpot assays were performed as previously described [49] with minor modifications. Briefly, Polyvinylidene difluoride-backed 96-well microtiter plates (MAIPS4510; Millipore Sunnyvale, CA, USA) were coated overnight at 4°C with 100 μl/well of specific capture antibody anti-IFN-γ or anti-IL-10 (10 μg/ml, 100 μl/well, Endogen Woburn, MA) dissolved in phosphate buffered saline (PBS).

Ab-coated plates were then washed three times and incubated 1 h at 37°C with complete medium to prevent non-specific protein binding. PBMC were plated at 3.5 × 10^5^/well, to a final volume of 200 μl/well of complete medium. Three different concentrations of NiSO_4 _(5–10–20 μg/ml), PdCl_2 _and Rh(CH_3_COO)_2 _(2.5–5–10 μg/ml) were added in triplicate wells and plates were kept in a humidified atmosphere with 5% CO_2 _at 37°C. Control triplicate wells contained PBMC with medium alone or with phytohaemagglutinin (PHA 1 μg/ml, Sigma, Saint Louis, Missouri, USA). After 48 hrs for IFN-γ and 72 hrs for IL-10 detection, cells were lysed with ice-cold distilled water, and cells were removed by 4 rinses with PBS/0.05% Tween^® ^20 (Sigma, St Louis, MO, USA). After a 90 min incubation with biotin labeled anti-IFN-γ or anti-IL-10 detection antibodies (1 μg/ml, 100 μl/well, Endogen Woburn, MA), HRP-Streptavidin-alkaline phosphatase (Euroclone, PBS 1% BSA) was added to the wells at a dilution of 1:1000 for 30 min at 37°C in the dark. Subsequent to specific substrate addition, the red spots were developed using AEC-chromogen solution (Sigma, St Louis, MO, USA) and analysed by the Automated ImmunoSpot Image Analyzer Software (AELVIS Tecnologies, TEMA ricerche, Italy). The results were expressed as the ratio (Index) between the number of spot forming cells detected upon stimulation and those present in the absence of stimuli. A positive response is defined by an Index > 3.

### Statistical analysis

The Mann-Whitney (MW) rank sum test was used to compare the cytokine responses between two different groups of patients. To evaluate the correlation between IFN-γ and IL-10 producing cells, the Spearman rank coefficient r was considered. A P value < 0.05 was considered to be statistically significant. Statistical analysis was performed with GraphPad Software, version 4.00 (San Diego California USA).

## Authors' contributions

VB: conceived of the study and participated in its design, carried out the ELISpot assays, drafted the manuscript. FP: carried out the patch tests and helped in the draft of the clinical section of the manuscript. PC–F: provided writing assistance and participated in the design and coordination of the study, performed the statistical analysis. AV: cryopreservation of PBMC from peripheral blood of patients enrolled in the study, carried out the Limulus assay on metal salts used for in vitro cytokine assay. AA: performed the statistical analysis and set the database with patients' clinical information. MP: carried out the patch tests, participated in the design of the study. FE: carried out the patch tests, provided writing assistance, participated in the design of the study. AC: carried out the patch tests, participated in the design of the study and participated in its coordination. All authors read and approved the final manuscript.
